# Does the aura surrounding healthy-related imported products fade in China? ERP evidence for the country-of-origin stereotype

**DOI:** 10.1371/journal.pone.0216866

**Published:** 2019-05-23

**Authors:** Bonai Fan, Qianrong Zhang

**Affiliations:** 1 School of Public Affairs, Zhejiang University, Hangzhou, China; 2 China Non-Public Economy Research Base, Ningbo University, Ningbo, China; Daegu University, REPUBLIC OF KOREA

## Abstract

Chinese consumers’ craze about imported products, especially foods and drugs, peaked after various safety incidents, such as the contamination of Chinese dairy products. Recently, this boom has gradually receded because of the constant quality problems of imported products and the stricter safety supervision of domestic products. Researchers have measured consumer’s perception toward domestic and imported products in various ways. In the current research, we investigated whether the country-of-origin stereotype has weakened in Chinese young consumers at the neurological level. By using a word-pair paradigm, 21 young participants were required to classify positive or negative words while event-related potentials were recorded. The results showed that reaction time to identify negative words following presentation of imported products (imported-negative condition) was longer than domestic products (domestic-negative condition). The amplitudes of N270 and LPP evoked in the imported-negative condition were significantly larger than those in the domestic-negative condition, possibly reflecting the higher expectation conflict when participate identified the adjectives as negative primed by imported healthy-related products. These findings revealed that young Chinese consumers still evaluated imported products better than domestic products.

## Introduction

With the development of economic globalization, consumers are exposed to a wider range of foreign products than ever before, which also brings a more competitive market for companies. Hence, the search to understand consumers’ perceptions and evaluations of domestic and imported products has become exceedingly important to business practitioners, country policy makers and academic researchers. As the world's second largest economy, the attitude of Chinese consumers, especially younger generations, toward domestic and imported products is critical to the world market.

Consumers’ perception of foreign and domestic products is one of the oldest concerns of international marketers. Since Schooler, the Country-of-Origin (COO) effect has been a continuing concern to marketing science [1]. The COO effect refers any influence or bias that consumers may hold resulting from the COO of a product [2–4]. Numerous empirical studies have shown that products from developed countries are more popular, while those from developing countries tend to receive a lower evaluation [5–7]. This was particularly obvious in developing countries. Most researchers explained this phenomenon from the perspective of information processing. They claimed that consumers’ experience with and storage of information about products and origin of products in the past was the key to their evaluation of the products. As the information asymmetry between buyers and the sellers of the product increases, the consumer would evaluate a product based on the clues available. COO is an easily recognized extrinsic cue that can be used as a signal for overall product quality and quality attributes [8]. When lacking other explicit information, the country image exerts a tremendous influence [9–11]. If a consumer had a good view of a specific country, he or she might have a preference for the products made by that country. The halo effect made consumers overgeneralize and made that idea a reality in their mind [12].

Another explanation is about emotion. In a survey of midwestern US consumers' preferences for Korean restaurants, researchers found that the cognitive image of that nation affected consumer attitudes from an emotional point of view [13]. Nationalism might affect, and even dominate, consumers’ purchasing behavior [14, 15]. Shimp and Subhash developed the CETSCALE (Consumer Ethnocentrism Tendency Scale) and found that the scores for the scale had a strong negative correlation with consumers’ attitudes and intention to purchase foreign products [16]. In addition, a large number of studies on consumer hostility showed that hostility negatively affected product evaluation and buying intention [17, 18].

However, some recent studies questioned the role of the COO effect. Globalization brought the deepening of the international division of labor, as well as the proliferation of production and distribution technologies, which decreased the role of COO as the “signal” of quality [19]. Second, consumers were hardly able to distinguish the correct origin country of the products or brands [20–22, 3]. Additionally, there was evidence that the COO effect in consumers in developed countries declined because of the ambiguity of the product sources [23], the maturity of consumers and the standardization of products [24]. However, in developing countries, the COO effect remained significant. Products from developed countries were given the meaning in relation to fashion, modernity, safety and so on [25,26].

Chinese consumers’ attitudes seem more complicated. In recent years, vibrant oversea shopping has reflected Chinese consumers’ preferences for foreign products. Especially after the Sanlu milk powder incident, the craze for foreign products, especially food and drugs, took off. However, with economic development and technological upgrades, consumers are constantly changing their attitudes toward domestic products. Some early research declared that Chinese consumers liked foreign products rather than domestic products in almost every category [27,28]. Recent studies, however, show that foreign products no longer have an absolute advantage relative to domestic products [29]. This is particularly true for young consumers who were born after the 1990s and have grown up in the rising process of the country’s enterprises. They face a market of both glamorous imported products and good-quality domestic products. They have experienced many food and drug safety incidents and have access to more comprehensive information about imported products, such as the recall of Hochwald milk. Under the impact of all of this information, is the influence of the COO effect weakened and has the beautiful aura surrounding imported products faded today? This is the question we attempt to answer in the current research. Considering the healthy-related products like food and drugs are the products that consumers are most exposed to, we choose healthy-related products as the research object in the study. Consumers are sensitive to the information about the healthy-related products. Consumers’ evaluation of food and drug is highly likely to expand into an evaluation of all products in a country because of the halo effect.

Previous studies on the COO effect mostly focused on the product image of specific countries, while they have seldom focused on the perspective of imported and domestic product images. Balabanis and Diamantopoulos proposed the concept of “foreignness” and “domestic country bias (DCB)”[21]. However, that paper only discussed why consumers in developed countries preferred domestic products rather than imported products. For developing countries, the situation of domestic bias may be different. It is in this sense that we broke the conceptual restrictions of COO as a single national area and expanded to imported products, which was a concept beyond a pure geographical meaning. It may better reflect the social and economic development level of a country.

In addition, regarding research methods, subjective methods and surveys cannot completely reflect a consumer's real feelings. These methods were self-reported and could not be supervised. In addition, numerous factors, such as time pressure, memory ability and incentives, motivated participants to distort the reporting of their feelings [30]. The evidence was simple that COO cues affect consumers on some emotional level [31,32]. There was still no scientific evidence, however, showing that COO information indeed activates quality perception, and the underlying neurophysiological explanation for this effect remains unknown. Hence, more accurate methods were needed to measure the user's inner intention, even if it was unconsciously formed.

Min et al. debuted the traditional method and used electroencephalography (EEG) to assess brain activities associated with the COO effect and consumer evaluation of product design [33]. Their study showed that the later P500 component reflected the cognitive assessment of COO in the associative parietal cortex. Based on these results, the researchers concluded that “COO influences product design preference”, which was part of a consumer’s affective feelings toward a product.

Thus far, research on the effect of COO with a neurophysiological approach has been insufficient. Here, we tried to enrich the COO effect research with this new method. The COO effect is essentially a kind of stereotype. Previous ERP studies on stereotypes and prejudice could help us to research this topic.

Most studies on stereotypes have been based on the hypothesis that stereotypes are a special semantic connection stored in long-term memory [34–36]. The semantic method is a common paradigm in researching stereotypes and prejudice [37–40]. In the current study, we also adopted the semantic method to capture consumers’ attitudes toward domestic and imported products. Many previous studies have found that the conflict between semantic information and expectations would elicit later positive potentials (LPP) [38,41,42], typically occurring in the interval starting at 300–400 ms and ending at 900 ms [43,44]. In addition, N400 has also been interpreted as an indicator to measure stereotypes [39,40]. However, Previous fMRI study showed that cognitive processing process of person judgment was distinctive from that of products judgment [45]. The cognitive monitoring mechanism of stereotype in product evaluation may be more sensitive than in person evaluation, which will reflect on N270 rather than N400 [46]. The N270 and N400 essentially play the similar cognitive monitoring role [47], but reflect the time at witch properties of target stimulus are compared with the working memory representation [48]. N270 was suggested to be related to conflict processing [49,50], mismatch of numbers [51], color [52], shape [53] and semantics [54] all trigger N270. Especially, Ma et. al. found N270 could be evoked by the conflict of lexical content on product judgment [55]. Xie et al. found N270 reflected the cognitive monitoring and behavioral control in country-of-origin stereotype-based decision making[46].

According to the aforementioned findings, we hypothesized that in the current study, consumers may present different brain activity, manifested in N270 and LPP amplitudes, while distinguishing positive and negative words. If the stereotype of imported healthy-related food was positive, the N270 and LPP elicited by negative adjectives following imported products will be larger than following domestic products and larger than the N270 and LPP elicited by positive adjectives following imported products. Logically for the converse, If the stereotype of domestic healthy-related food was negative, the N270 and LPP elicited by positive adjectives following imported products will be larger than following imported products and larger than the N270 and LPP elicited by negative adjectives following domestic products.

## Methods

All methods used in this experiment were in accordance with the relevant guidelines and regulations. All participants provided written informed consent before the experiments began and received 40 yuan after the experiment as reward. The current study was approved by the Internal Review Board of the Academy of Neuro-economics and Neuro-management at Ningbo University.

### Participants

Data were collected from 24 healthy right-handed young adults (12 male and 12 female) aged 19–26 years (M = 22.04, SE = 1.97) who provided consent to participate in this experiment. All the participants were Ningbo University students and had experience buying imported products. Additionally, all the participants claimed to have not much preference toward imported products, and the result of CETSCALE (Consumer Ethnocentrism Tendency Scale) showed no significant consumer ethnocentrism tendency in these participants. Three subjects’ data were discarded for excessive ERP artifacts; thus, 21 (10 male) valid subjects were included in the final data analysis.

### Materials

The stimuli consisted of 96 word pairs (12 nouns × 8 adjectives) that were repeated once. The word pairs were divided randomly into 4 blocks. The first word (Stimulus 1, the prime stimulus) was one of 12 selected products, 6 imported products and 6 domestic products. To understand people’s attitude toward health-related products, we choose milk, milk powder, beef, vaccine, calcium and antibiotic as the prime stimulus words. The second word in each pair (Stimulus 2, the target stimulus) was a four-word Chinese adjective which was usually be used to describe the products (positive or negative).

To construct a set of second stimulus words, we chose 20 four-word Chinese words (half positive and half negative) that can describe food and drugs. Thirty students (the 24 EEG participants were not included) rated the 20 adjectives for their suitability to describe imported and domestic food and drugs. If the stereotype about one of the two groups was negative, the rated results for the negative adjectives were taken into account to select the negative words. The same method was used for selecting positive words. A five-point Likert-type scale was used to evaluate the suitability. Eight words above the average score of 4.14 were selected as target stimuli. The negative adjectives included the Chinese terms for shoddy, counterfeit, jerry, rough; the positive adjectives included the Chinese terms for reliable, upmarket, high-quality, genuine.

The 128 stimuli for prime-target pairs were divided into four conditions: imported product-positive word (IP); domestic product-positive word (DP); imported product-negative word (IN); and domestic product-negative word (DN). Each Chinese word was made into a picture and digitized at 360×270 pixels. Stimuli were presented sequentially in the center of a computer screen with a visual angle of 2.58°×2.4°.

### Procedure

**Fig 1:**
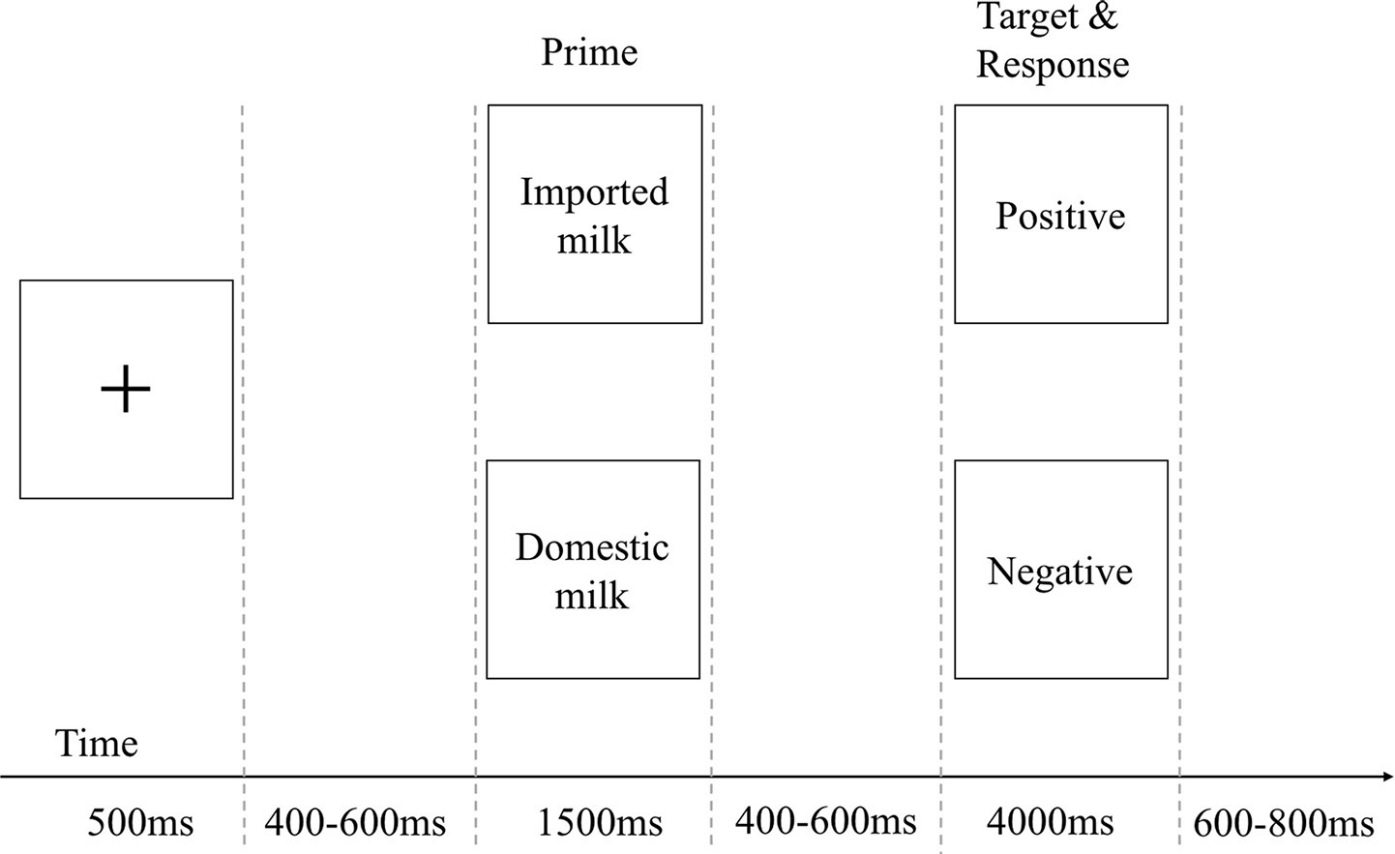
Schematic of the word classification task.

Each participant was seated in a chair 1 m from a computer monitor. The experimenter informed participants that they would see a series of word pairs and that their task would be to categorize the second word as positive or negative as quickly as possible. The 192 stimulus pairs in the entire experiment were randomly distributed into 4 blocks with 48 trials each. The 48 word pairs in each block were presented randomly. Each trial began with a pattern mask (500 ms), followed by the prime (1500 ms) and then the target. Each participant was asked to give his/her response within 4000 ms of the target word onset. The interval between the end of the previous Stimulus 2 and the onset of the following Stimulus 1 was 600–800 ms (see Fig 1). Each participant rested for 2 min before the next block began. Stimuli and recording triggers were presented using Curry 7.0 software.

In the trials, the prime consisted of a noun (either “domestic product" or “imported product”), and the target consisted of either a positive word or a negative word (e.g., reliable, shoddy).

### EEG recording and analysis

Electroencephalogram (EEG) was recorded continuously (bandpass fliter 0.05–100 Hz, sampling rate 500 Hz) in conjunction with Curry 7.0, using an electrode cap with 64 Ag/AgCI electrodes mounted according to the extended international 10–20 system and referenced to digital, linked mastoids. The vertical electrooculogram was recorded from the right eye by supra-orbital and infra-orbital electrodes. The horizontal electrooculogram was recorded from electrodes on the outer canthi of both eyes. All EEG electrode impedances were maintained below 5 kΩ. Electroencephalogram recordings were segmented for the epoch from 200 ms before the onset of appearance of the target word to 800 ms after this onset with the first 200 ms before the target as a baseline. Electroencephalogram artifacts were corrected using the method proposed by Semlitsch et al [56]. Trials contaminated by amplifier clipping, wherein bursts of electromyography activity and peak-to-peak deflection exceeded ±80 μV were excluded. The EEG recording for each recording site for every participant was averaged separately within each of the experimental conditions. Then, averaged ERPs were digitally filtered with a low-pass filter at 30 Hz (24 dB/octave).

Based on the visual inspection of the grand-average waveforms the method proposed by Picton et al.[57], two ERP components were analyzed: N270 and LPP. For N270 component, a time window of 290–350 ms and nine electrode sites- F1, FZ, F2, FC1, FCZ, FC2, C1, CZ, C2-were chosen for data analysis in the basis of pervious neuroscience findings [49, 55]. For LPP component, a time window of 530–650 ms and six electrode sites-C1, CZ, C2, CP1, CPZ, CP2-were chosen for data analysis [58]. To examine the effects of caudality ad laterality, as well as the anchor condition, repeated-measures ANOVAs were performed to assess the effects of four factors, product (imported, domestic), adjectives (positive, negative), caudality (anterior to posterior), and laterality (left, middle, right), on the two components. Simple effect analyses were conducted when interactive effects appeared, and data were corrected according to the Greenhouse–Geisser method when necessary, and multiple comparisons were corrected with the Bonferroni method when appropriate.

## Results

### Behavioral data

**Fig 2:**
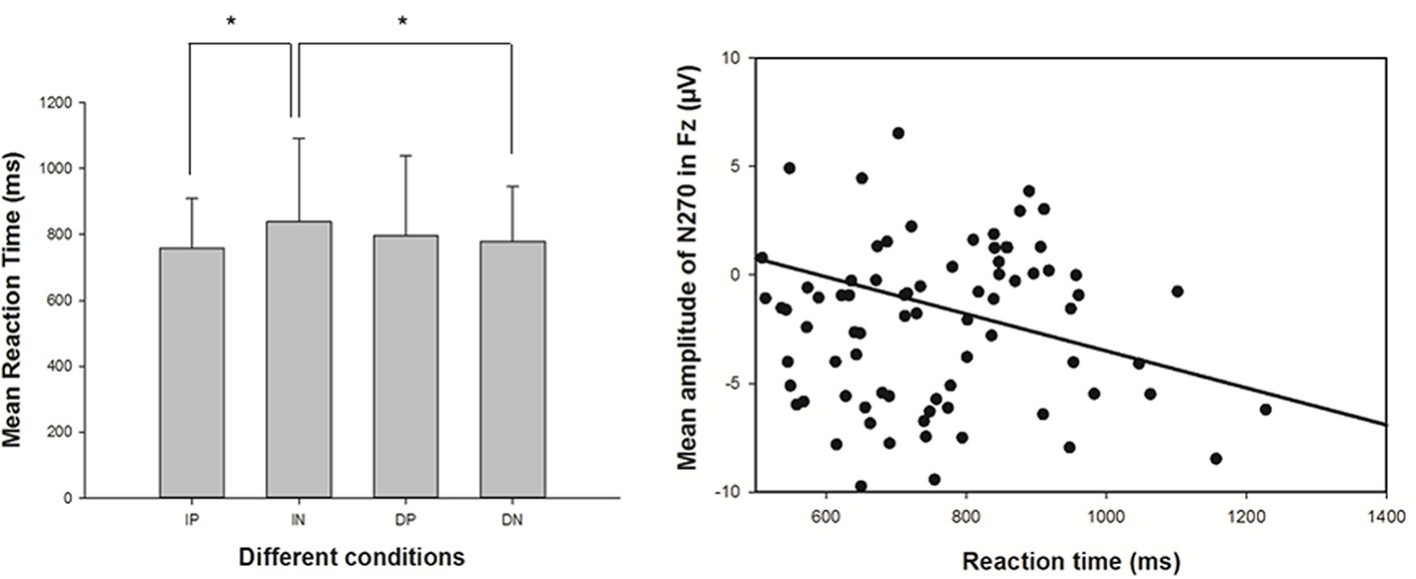
Behavioral result of the participants. (**a)** The reaction time (RT) under four conditions. (b) Correlation between the amplitude of N270 on the Fz electrode and the reaction time.

Behavioral data are shown in Fig 2(A). A 2 (imported/domestic product) × 2 (positive/negative word) ANOVA showed that there was no significant main effect for the products (F[1, 20]) = 0.783, p = 0.387; η^2^ = 0.038). However, there was a significant main effect for the adjectives (F[1,20] = 4.456, p = 0.048; η^2^ = 0.182). A marginally significant interaction effect between products and adjectives (F[1,20] = 3.744, p = 0.067; η^2^ = 0.158) was been observed. Simple effect analyses revealed that there was no significant effect of products in reaction time when connected to positive adjectives(F[1,20] = 1.817, p = 0.193; η^2^ = 0.083)(imported-positive: M = 757.18ms. SE = 152.05; domestic-positive: M = 796.53ms. SE = 242.20). However, participants identified negative adjectives following domestic products (M = 779.43ms, SE = 166.95) significantly quicker than those following imported products (M = 839.57ms. SE = 251.16) (F[1,20] = 4.850, p = 0.040; η^2^ = 0.195). The participants also identified positive adjectives more quickly than negative adjectives following imported products (F[1,20] = 5.403, p = 0.031; η^2^ = 0.213) whereas no significant effect was observed when identified positive and negative adjectives following domestic products(F[1,20] = 0.537, p = 0.472; η^2^ = 0.026). These results were similar to the results of previous studies on stereotypes and implicit attitudes or metaphors [40,59,60]. Although the participants were self-avowed egalitarians and neutral, the time they spent categorizing different objects was significantly different.

### ERP results

#### N270

**Fig 3:**
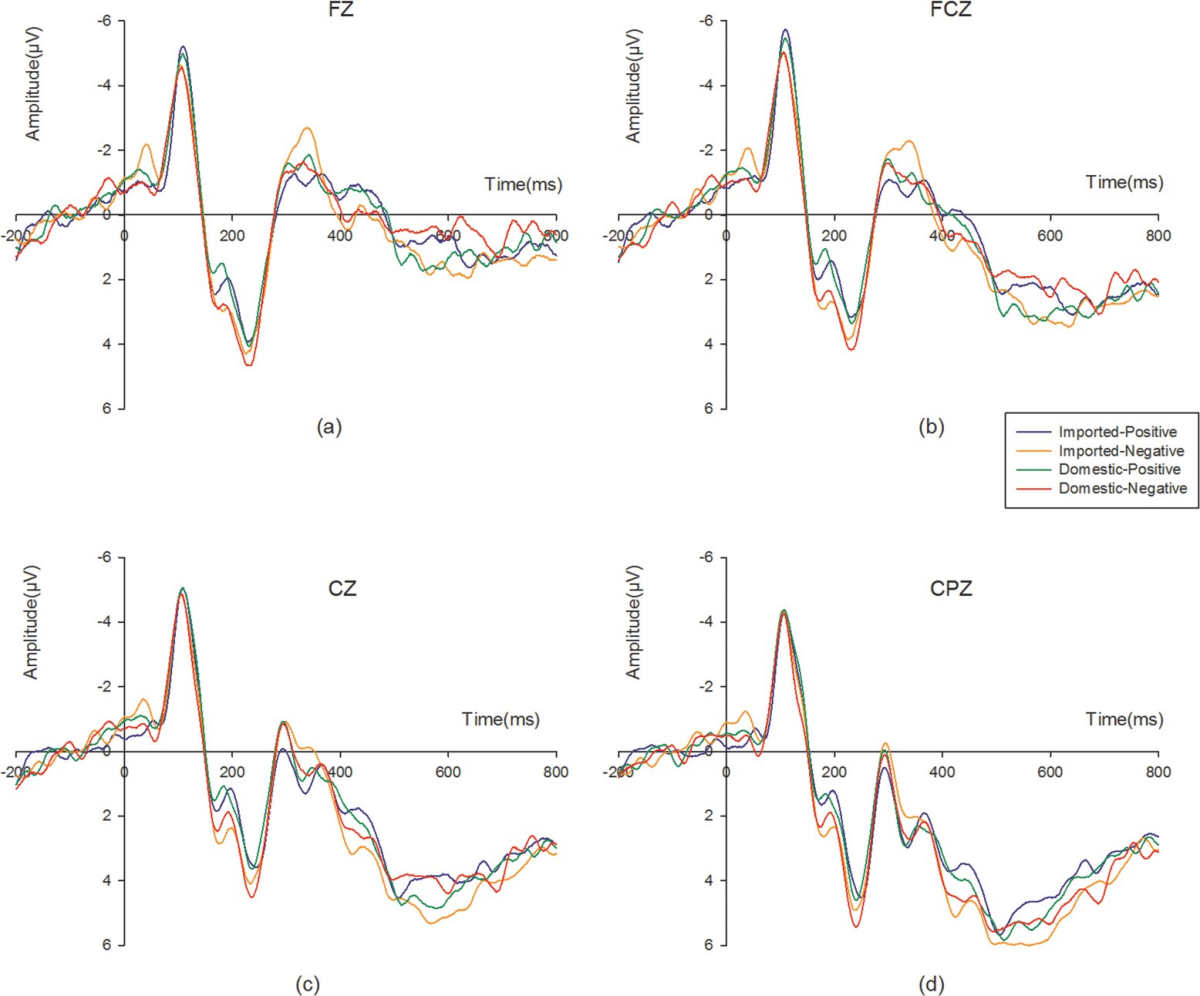
Grand-average ERP wave forms under the four conditions. (a) N270 amplitude on FZ. (b) N270 amplitude on FCZ. (c) N270 and LPP amplitude on CZ. (d) LPP amplitude on CPZ.

As shown in Fig 3(A), 3(B), 3(C), the four-way 2 (product: imported, domestic:) × 2 (adjectives: positive, negative) × 3 (caudality: F, FC, C) × 3 (laterality: left, middle, right)ANOVA for N270 produced no significant main effects for product (F[1,20] = 0.004, p = 0.948; η^2^ = 0.000) and adjectives (F[1,20] = 2.552, p = 0.126; η^2^ = 0.113). However, both factors of electrode distribution had significant effects on the N270 amplitude (caudality: F[2,19] = 10.062, p = 0.001, η^2^ = 0.514; laterality: F[2,19] = 6.111, p = 0.009, η^2^ = 0.391). The post hoc pair-wise comparisons with Bonferroni correction indicated that the N270 was significantly larger in the front and front central than the central regions (p _FC,C_ = 0.051; p _F,C_ = 0.002). But there was no significant disparity between front and front central region (p _F,FC_ = 1.000). The effect of laterality indicated the the N270 in the middle was larger than in the left and right hemisphere (p _middle,life_ = 0.038;p_middle right_ = 0.067). However, there was no significant disparity in the N270 between the left and right hemispheres (p_left, right = 1.000_). Thus, the amplitude was lager in the front region and the midline and reached a maximum at Fz.

The interaction effect of product and adjectives was significant (F[1,20] = 5.645,p = 0.028; η^2^ = 0.220). Simple effects tests showed that there was no significant effect for product when adjective was fixed as positive (F[1,20] = 2.093, p = 0.164, η^2^ = 0.095) (imported-positive: M = -0.056μV, SE = 4.285; domestic-positive: M = -0.681μV, SE = 4.609), whereas there was a significant effect when adjective was fixed as negative (F[1,20] = 2.342, p = 0.139, η^2^ = 0.089). The amplitude of N270 was much larger for the pair imported-negative (M = -1.226μV, SE = 5.056) than for the pair domestic-negative (M = -0.568μV, SE = 4.961). Moreover, there also no significant effect for adjective (F[1,20] = 0.060, p = 0.820; η^2^ = 0.003) when the product was fixed as domestic. However, the effect of adjective was significant (F[1,20] = 9.042, p = 0.007; η^2^ = 0.311) when the products was fixed as imported, and the amplitude of N270 were larger to the pair imported-negative than domestic-negative.

Additionally, an interaction effect among product, adjective and laterality was been observed (F[2,19] = 6.939, p = 0.005, η^2^ = 0.422). Further simple effects analysis demonstrated that the N270 for the pair imported-negative and domestic-negative was significantly different in the midline(F[1,20] = 4.661, p = 0.043; η^2^ = 0.189) and on the right hemisphere(F[1,20] = 7.753, p = 0.011; η^2^ = 0.279). The N270 for the pair imported-negative and imported-positive was also significantly different in the midline (F[1,20] = 8.258, p = 0.009; η^2^ = 0.292) and on the right hemisphere(F[1,20] = 14.251, p = 0.001; η^2^ = 0.416). No significant difference was found on the left hemisphere (F[1,20] = 2.902., p = 0.104; η^2^ = 0.127).

No significant effects were found between product and caudality (F[2.19] = 0.104, p = 0.902.; η^2^ = 0.011), between adjective and caudality (F[2,19] = 2.220, p = 0.136, η^2^ = 0.189), between product and laterality (F[2,19] = 0.585, p = 0.567, η^2^ = 0.058), between adjectives and laterality(F[2,19] = 0.104, p = 0.902, η^2^ = 0.011) as well as between caudality and laterality (F[4,17] = 1.425, p = 0.268, η^2^ = 0.251) There was no three-way interaction effect between product, adjective and caudality(F[2,19] = 0.591, p = 0564, η^2^ = 0.059), between product, caudality and laterality(F[4,17] = 0.127, p = 0.971, η^2^ = 0.029) and between adjective, caudality and laterality(F[4,17] = 0.829, p = 0.525, η^2^ = 0.163). The four-way interaction effect was not observed (F[4,17] = 0.122, p = 0.973, η^2^ = 0.028).

Finally, we conducted a Spearman correlation analysis of the reaction time and the mean voltage of N270 on the FZ electrode. As shown in Fig 2(B), there was a highly significant correlation between reaction time and the mean voltage N270(r = -0.325. p = 0.002). Thus, the reaction time increase with an increase in the N270 amplitude (negative polarity: smaller voltage value means larger amplitude) in this experiment.

#### LPP

Fig 3(C) and 3(D) showed the ERP waveforms for each electrode site under the four stimulus conditions. We also conducted the four-way 2 (product: imported, domestic:) × 2 (adjectives: positive, negative) × 2 (caudality: C, CP) × 3 (laterality: left, middle, right) ANOVA. There was no significant main effect for either products (F[1,20] = 0.637, p = 0.434; η^2^ = 0.031) or adjectives (F[1,20] = 1.159, p = 0.295; η^2^ = 0.055). However, there was a marginally significant main effect for caudality (F[1,20] = 4.022, p = 0.059, η^2^ = 0.167) and a significant effects for laterality(F[2,19] = 5.076, p = 0.017, η^2^ = 0.348). The post hoc pair-wise comparisons with Bonferroni correction indicated that the LPP was marginally significantly larger in the central posterior than central regions (p_CP,C_ = 0.059). The effect of laterality indicated the the LPP in the middle was larger than in the left hemisphere (p _middle,life_ = 0.012). However, there was no significant disparity of LPP between the left and right hemispheres (p_left, right = 0.253_) and between the middle and right hemisphere (p_middle, right_ = 0.456). Thus, the amplitude was lager in the central posterior and the right hemisphere and reached a maximum at CPz.

Moreover, a significant interaction effect between products and adjectives was also observed (F[1,20] = 4.906, p = 0.039; η^2^ = 0.197). Simple effect analysis results showed that there was a marginally significant effect for the product when adjective was fixed as negative (F[1,20] = 3.484, p = 0.077; η^2^ = 0.248). The LPP amplitude elicited by the pair imported-negative (M = 4.895μV, SE = 4.132) was larger than the amplitude for the pair domestic-negative (M = 4.056μV, SE = 4.775). There was also a significant effect between the pair imported-negative and pair imported-positive (M = 3.948μV, SE = 4.491) (F[1,20] = 4.791, p = 0.041; η^2^ = 0.193). However, no significant difference for LPP was observed between the pair domestic-positive (M = 4.262μV, SE = 4.531) and the pair, the pair pair imported-positive as well as the pair imported-positive and the pair domestic-positive.

No significant effects were found between product and caudality (F[1.20] = 0.525 p = 0.477.; η^2^ = 0.026), between adjective and caudality (F[1,20] = 0.663, p = 0.425, η^2^ = 0.032), between product and laterality (F[2,19] = 1.064, p = 0.365, η^2^ = 0.101), between adjectives and laterality(F[2,19] = 0.462, p = 0.637, η^2^ = 0.046) as well as between caudality and laterality (F[2,19] = 0.093, p = 0.911, η^2^ = 0.010) There was no three-way interaction effect between product, adjective and caudality(F[1,20] = 0.112, p = 0.741, η^2^ = 0.006), between product, adjective and laterality (F[2,19] = 1.320, p = 0.291, η^2^ = 0.122), between product, caudality and laterality(F[2,19] = 0.800, p = 0.464, η^2^ = 0.078), between adjective, caudality and laterality(F[2,19] = 1.766, p = 0.198, η^2^ = 0.157). The four-way interaction effect was not observed(F[2,19] = 1.063, p = 0.365, η^2^ = 0.101).

Additionally, we conducted a Spearman correlation analysis of the reaction time and the mean voltage of LPP on the CPZ electrode. NO significant correlation between reaction time and the mean voltage LPP (r = -0.158. p = 0.151) was found.

## Discussion

The present study used a word-pair paradigm to identify brain mechanisms and ERP components underlying the stereotypes regarding imported products. The statistical results of N270 and LPP components showed an interaction effect between product and adjectives Further analysis showed that, connected to negative adjectives, the imported products elicited larger N270 and LPP amplitudes than domestic products, whereas no significant effect of N270 and LPP was observed when connected to positive words.

Consistent with previous behavioral research on stereotypes [61,62], the mean RT for the imported-negative condition was longer than that for the imported-positive condition and domestic-negative condition. This indicated that the participants might have confronted conflict and needed more time to process these conflicts when identifying the words of imported products and the negative words, which showed their automatic evaluation or the subliminal activation that the imported products were harder to relate to negative figures.

The N270 amplitude were larger in the stereotype-incongruent word pairs (imported-negative) than stereotype-congruent (imported-positive; domestic-negative) word pairs. According to previous ERP studies, N270 can be used as in index to information conflict [50, 52, 55] and N270 is sensitive to the conflict strength [63]. In the present study, imported-positive word pair was congruent with COO stereotype in consumer long-term memory that imported product is good and safe. Thus, more information conflict was triggered and larger N270 was elicited in imported-negative word pairs. Furthermore, the N270 difference was more lateralized to the right but not the left hemisphere. Previous research indicated that right dorsolateral prefrontal cortex (DLPFC) were more active on mismatch trials [64], which was in line with evidence of a conflict-monitoring system in the ACC acting in association with a DLPFC implementing cognitive control [65]. Thus, the larger N270 in imported products compared to domestic products over the right hemisphere may indicate more incoherent when related to negative adjectives. This interpretation is supported by a study that demonstrated greater activation in the right hemisphere on conflict condition [50]. Moreover, we found the N270 was positively correlated with the reaction time. These correlations suggest that the brain responses were consistent with the behavior. It implies that stereotype-related N270 may also connected with behavioral control. The analysis of the LPP showed that compared with domestic products, imported product elicited larger LPP amplitudes when classifying negative words. This finding is consistent with previous studies that have shown that LPP mainly reflects processing associated with expectancy violations [38, 39, 66]. Additionally, the dipole source analysis suggested that the LPP was located approximately in the ACC, which has been linked to errors and the monitoring of conflict [67]. Participants might need more effort at inconsistency resolution when expectations for imported products were positive. Therefore, we suspected that Chinese youth consumers had an expectation that imported products would be safer, healthier and of higher quality. In addition, LPP is an important late component of emotional modulation, which indicates the enhanced encoding of emotional words and expressions [63, 68]. LPP amplitude induced by high arousal stimulation was larger than that of low arousal stimulation [69]. In the present study, imported products with negative information elicited a larger amplitude of LPP component than domestic products. It indicated that imported products related to negative information aroused more intense emotion than imported products. As imported products were usually considered good, negative information was contrary to consumer’s cognition. The expectancy violations may have resulted in a high-level emotion arousal among consumers.

However, in our research, we did not find significant differences in the N270 and LPP amplitude between imported-positive and domestic-positive stimulus pairs or between domestic-positive and domestic-negative stimulus pairs. This might have reflected Chinese youth consumers’ attitudes toward domestic products were amphibolous. A possible reason was the recent positive propaganda in the mainstream media concerning “made-in-China” products as well as the better supervision on product safety. In addition, according to the results of the IN and DN stimulus conditions on N270 and LPP, it was obvious that in the early stage, the target attribute words have a significant effect on N270, whereas such an effect faded in the later and more complicated semantic processing. This might suggest that negative information influences consumers’ attention at first, but after deeply thinking, for example, the recall of the food and drug safety incidents of imported products, they would change their stereotypical view of imported products.

## Conclusion

Electrophysiological measurements offer a complementary approach to behavioral measurements for understanding young Chinese consumers’ attitudes toward domestic and imported healthy-related products. In the current study, we put forward two sensitive components, N270 and LPP, to judge the consumer’s attitude. The significantly larger N270 and LPP amplitude elicited by imported-negative word pairs compared with domestic-negative word pairs showed the positive aero surrounding imported healthy-related products was still existing. However, the result that there was no significant difference between IP and DP, DP and DN conditions might show a neutral attitude tendency toward domestic products with the efforts of supervision on product safety.

## Supporting information

S1 File Zip file containing raw experimental data. (ZIP)
